# Knowledge, Skills, and Compliance of Nurses Related to Central Line-Associated Bloodstream Infection in the Cardiovascular Department at King Faisal Hospital and Research Centre, Riyadh

**DOI:** 10.7759/cureus.30597

**Published:** 2022-10-23

**Authors:** Vanaja Perumal, Yasser Abdulrhman Alheraish, Muhammad Shahzad, Siti Maarof, Mavic Perez, Pradeep Nair

**Affiliations:** 1 Cardiac Surgery, King Faisal Specialist Hospital and Research Centre, Riyadh, SAU; 2 Critical Care, King Faisal Specialist Hospital and Research Centre, Riyadh, SAU; 3 Nursing, King Faisal Specialist Hospital and Research Centre, Riyadh, SAU

**Keywords:** clabsi, hai, skills, attitude, knowledge

## Abstract

Background and objective

Healthcare-associated infections (HAIs), especially central line-associated bloodstream infections (CLABSI), are among the most critical public health problems worldwide. Knowledge, attitude, and skills of nurses are vital in HAI prevention. In this study, we aimed to assess nurses' knowledge, skills, and compliance related to CLABSI.

Method

This study was conducted in a heart center as a prospective interventional study. Eighty nurses were selected after obtaining their consent to participate in the pretest, posttest, and skills review. Qualified nurses registered with the Saudi Council and working for at least one month in the relevant unit at the time of the study were included. Nurse managers, interns, and student nurses were excluded. Nurses' skills were analyzed using a competency-based checklist approved by the hospital.

Results

We enrolled 80 participants in our study. The majority of the participants (51.25%) fell under the age group of 25-34 years. There were 68 females (85%). Participants with an experience of 6-10 years constituted the biggest proportion (37.5%) in the cohort in terms of work experience. The mean CLABSI knowledge-related pretest and posttest scores were 6.7 ±1.09 and 6.8 ±1.11, respectively, while the CLABSI compliance scores were 8.1 ±0.99 and 8.3 ±0.97, respectively.

Conclusion

Based on our findings, clinical experience of more than five years is associated with good CLABSI knowledge and compliance among nurses. Nurses' level of education also had a significant relationship with CLABSI pretest and posttest scores.

## Introduction

Healthcare-associated infections (HAIs) are among the highly critical hospital problems worldwide [1]. A frequent source of HAI is central line-associated bloodstream infections (CLABSI) where organisms, usually bacteria, enter the bloodstream. Hyperosmolar medications, such as vasopressors, antibiotics, chemotherapy, and parenteral nutrition, are administered using central venous catheters (CVCs). Several risk factors for CLABSIs have been identified in cardiac patients, such as longer duration of indwelling catheters, bacterial colonization at the insertion site and the catheter hub, and insufficient care/maintenance of the CVC after insertion [2]. Among all HAIs, the incidence of CLABSI is around 9% [3]. CLABSIs can result in prolonged lengths of stay, high costs to the hospital, and an increase in morbidity and mortality [4]. Sometimes, line access may be challenging; each vessel presents unique risks and complications [5,6]. Effective handwashing practice with the Aseptic Non-Touch Technique (ANTT®) is vital in reducing the risk of infection and should be applied to all aspects of care and management [7].

Among healthcare workers (HCWs), nurses in cardiovascular settings have the most direct and regular role in performing high-risk CVC procedures. Therefore, they should be knowledgeable and compliant when assisting during CVC insertion as well as care and management of central lines [8]. Many surveys from various countries have provided information on the knowledge level, attitudes, and the degree to which evidence-based practices are utilized among nurses in other settings [9]. To ensure the appropriate use of CVCs, nurses' in-depth knowledge and documented competency of the issues are essential.

Therefore, education, skills, and nurses' compliance are critical priorities for reducing CLABSIs by implementing proper preventive measures in line with evidence-based recommendations. Practical-based competency involves skill performance or simulation of an actual patient situation to evaluate competence. Knowledge and skill evaluation can be assessed with hands-on demonstrations. In addition, it has been reported that nursing actions and compliance with best practices directly influence outcomes [10,11]. Nurses with inadequate knowledge may fail to provide appropriate care and maintenance for central lines and may inadvertently contribute to CLABSI incidence.

Our study aimed to assess the nurses' level of knowledge, skills, and compliance with CVC procedures. Furthermore, the study sought to examine if nurses' experience, age, and education level can affect their adherence to central line management. We tried to evaluate the effects of training on nursing practice in terms of knowledge, skills, and compliance related to central lines care and CLABSI rates.

## Materials and methods

As most cardiac patients have a central line for monitoring and medication administration, we conducted a prospective interventional study in the heart center. Approximately 180-200 staff nurses work in this center in six different sections. A convenience sampling method of the non-probability sampling design was used for selecting nurses as participants. Eighty nurses were selected after obtaining their consent to participate in the pretest, posttest, and skills review. We included qualified nurses registered with the Saudi Council who had worked for at least one month in the cardiac unit at the time of the study. We excluded nurses with less than one month of experience and floating nurses from other departments. In addition, nurse managers, interns, and student nurses were excluded as they had infrequent involvement with patient care. After explaining the study's purpose to participants, informed consent was obtained.

Every participant was informed that participation was voluntary and withdrawal was possible at any time. Furthermore, the respondents were assured that participation, withdrawal, or refusal to participate would not affect their entitlement to health services. Before signing the consent, there was a question-answer session to ensure that the participants fully understood the explanations. Following the briefing sessions, the respondents were asked to sign a consent form. The research project obtained approval from Institutional Review Board (IRB) (approval no. 2211173). Once permission was obtained, nurses in the heart center were invited via a notice put up at the nursing unit board and personal communication.

The goal of the educational intervention was to reduce catheter-related infection rates, and a before-and-after evaluation was planned to evaluate its effectiveness. A pretest and posttest design was adopted to analyze how well-versed are the nurses in central catheter care infection prevention and control practices. The target groups of 80 nurses working in the Cardiac ICU (adult and pediatric), Coronary Care Unit, Cardiovascular Step-down, and Cardiovascular Telemetry Unit were engaged in patient care with central lines. CLABSI was defined as a laboratory-confirmed bloodstream infection in a postoperative cardiac surgical patient where the mainline was in place for >48 hours with no other apparent source except the catheter. One or more positive blood cultures without other apparent source or clinical signs of infection, such as fever >38 °C, chills, or hypotension, with an organism unrelated to another infection site and the same organism cultured from two or more separate blood samples, were referred to as CLABSI. The timeframe between any two elements, like fever and positive blood cultures, was 24 hours (Warren et al., 2004). One catheter day was defined as patients having one or more catheters for more than 24 hours. The CLABSI rate was the number of catheter-related infections per 1000 catheter days [12].

All nurses were assessed using the following two criteria: a questionnaire for practical skill assessment, for compliance for six months, from June 2021 to December 2021. The questionnaire was developed based on the CDC Recommendations for the Prevention of Intravascular Catheter-Related Infections and was in English. Knowledge-related questions involved nurses' daily CVC management, such as a recommended policy to change parenteral nutritional lines and claves, cleaning the insertion site, and collecting a blood sample. Questions related to policy for dressing change, method of central line flushing, and knowledge of CLABSI definition were also included. Compliance-related questions focused on the CVC bundle, line maintenance, line assessment, chlorohexidine bath, and line documentation. The competency-based checklist consisted of four sections - section A: general CVC management; B: flushing the CVC through needleless adapter - ANTT standard; C: CVC dressing; and D: changing the needleless adapter and intravenous tubing.

Instruments

We designed the questionnaire, and the whole study was designed to have three parts. The first part involved the nurses' demographic and professional characteristics (age, gender, educational level, years of experience, unit of activity, single skill or varied skills, and shift coverage).

The second part evaluated nurses' knowledge of evidence-based practices. The choices for the responses were "yes," "do not know," and "no". The number of correct answers was calculated and entered into SPSS Statistics version 23 (IBM Corp., Armonk, NY). The third part included questions measuring compliance with guidelines. Ten questions were scored using a 3-point Likert-type scale with options for "agree," "uncertain," and "disagree". The correct answers were scored and documented. A score below 5 was considered low, 6-7 was medium, and a score of 8-10 was considered high. The questionnaire was pretested to evaluate the questions' clarity and reliability. The final version of the questionnaire was refined and corrected based on feedback from the participants. The participant's involvement was kept anonymous to encourage participation and reduce bias. Likert-scale questionnaires are the most commonly used instrument for measuring variables such as motivation and self-efficacy. They enable the researchers to assemble large amounts of data with relative ease. However, despite their widespread use, there is relatively little information in the literature other than in English concerning the development and analysis of Likert-scale questionnaires.

Nurses' skills were assessed by using competency-based checklists approved by the hospital, including CVC dressing, CVC flushing, and clave change formulated by Nursing Development & Saudization (ND&S) (2020). In addition, nurses' compliance level was evaluated using this checklist, and the components were based on the policy and procedures of central line management. The competency was scored 1 for met and 0 for not met criteria. The competency-based checklist consisted of the following sections - A: general CVC management; B: consists of 20 criteria. Less than 15 criteria were marked as not met: flushing the CVC through a needleless adapter - ANTT standard (nine criteria), less than seven were not met; C: CVC dressing (17 criteria), less than 15 criteria were reported as not met, and D: changing the needleless adapter and intravenous tubing (11 criteria), less than nine criteria were reported as not met (Tables 1-4).

**Table 1:** General CVC management (met, not met) CVC: central venous catheter

Section A: standards for measuring the performance related to CVC care	
Displays effective verbal and non-verbal communication	
	a. Friendly and courteous/introduces self to patient/family/caregiver
	b. Professional
	c. Culturally sensitive, caring
	d. Educates patient and family/caregiver about procedures and obtains permission when relevant
1. Identifies patient by using two patient identifiers	
2. Verifies patient allergy status	
3. Verifies physician’s order for the procedure (when required)	
4. Assesses the patient’s general condition, lab values, and current medications	
5. Performs hand hygiene	
6. Places the patient in an appropriate, comfortable position ensuring easy access to the CVC relevant to the procedure	
7. Gathers supplies and dressing trolley	
8. Performs hand hygiene	
9. Dons non-sterile gloves	
10. Disinfects the dressing trolley	
11. Removes gloves and performs hand hygiene	
12. Wears personal protective equipment	
13. Sets up equipment using an aseptic technique	
14. Recognizes contamination during set-up and takes corrective action(s) if broken	
15. Maintains patient comfort and safety throughout the procedure	
16. Discards used supplies in the appropriate bin/sharps container	
17. Performs hand hygiene	
18. Documents in Electronic Medical Records	
19. Reports pertinent findings to a relevant physician	
20. Labels blood specimen tube(s)/containers and sends them to a laboratory in a timely manner: ID number, date, and time	

**Table 2:** Disconnecting infusion and/or flushing the CVC through a needleless adapter - ANTT standard CVC: central venous catheter; ANTT: Aseptic Non-Touch Technique

Section B: flushing the CVC through needleless adapter - ANTT standard
1. Performs hand hygiene
2. Dons non-sterile gloves
3. Selects the appropriate lumen for flushing
4. Removes the disinfectant cap or disconnects the line and places the dead-end male luer lock cap at the end of the IV tubing
5. Holds the lumen of the catheter utilizing ANTT. Does not lay the catheter down
6. Cleanses the adapter with a chlorhexidine/alcohol-impregnated wipe using friction and back-and-forth motion (top and sides) for 30 seconds. If the disinfectant cap in situ, does not cleanse the adapter
7. Allows to air-dry for at least 30 seconds
8. Attaches a prefilled syringe with 0.9% sodium chloride
9. Checks for blood return and then flushes the catheter with (peds: 3-5, adults: 10-20) ml 0.9% sodium chloride (adjusts for neonates)

**Table 3:** CVC dressing – ANTT CVC: central venous catheter; ANTT: Aseptic Non-Touch Technique

Section C: standards for CVC dressing change, with ANTT
1. Assesses the skin around the CVC dressing prior to any procedure
2. Dons a mask and plastic apron
3. Performs hand hygiene
4. Dons non-sterile gloves
5. Prepares the sterile field and opens supplies
6. Instructs the patient to turn their head away from the insertion site or mask the patient
7. Removes the old CVC dressing and securement device
8. Removes gloves
9. Performs hand hygiene
10. Dons sterile gloves
11. Places drape per location of CVC line
12. Performs a visual inspection of the CVC insertion entry/exit site and the surrounding area. Checks the sutures are intact (if applicable)
13. Cleanses the insertion site, around the insertion site, under the line, and down the line with appropriate skin disinfectant
14. Ensures the catheter is secured by using a securement device or Steri-Strips
15. Applies appropriate dressing by ensuring the site is covered and the dressing adheres to the skin. Follows manufacturer guidelines
16. Secures the tubing with a securement device if indicated
a. If the securement device is unavailable, place a Steri-Strip under the CVC sticky side up (below the dressing), wrap it around the catheter, and adhere it to the skin
b. Apply a small transparent dressing cut halfway through, and place the dressing under the catheter and at the bottom of the dressing to secure it
17. Labels the dressing with the date, time, and ID number

**Table 4:** Changing the needleless adapter/IV tubing – ANTT surgical ANTT: Aseptic Non-Touch Technique

Section D: standards for changing the needleless adapter with ANTT
1. Follows the steps in Section C 1-6
2. Performs hand hygiene. Dons sterile gloves
3. If not using sterile prefilled syringes of 0.9% sodium chloride, aseptically prepares two syringes of 0.9% sodium chloride
4. Primes the new needleless adapter with 0.9% sodium chloride
5. Holds the lumen of the catheter utilizing the "dirty hand" or nondominant hand technique
6. Ensures the catheter is clamped prior to opening and removing the adapter
7. Removes the old needleless adapter with 4x4 gauze and discards
8. Cleanses around the catheter hub sides with a chlorhexidine/alcohol-impregnated wipe for at least 30 seconds. Allows to air-dry. Does not go over the top of the open catheter
9. Attaches the pre-primed needleless adapter, aspirates for blood return, and flushes the catheter with (peds: 3-5, adult: 10-20) ml 0.9% sodium chloride (adjusts for neonates)
10. Clamps the catheter using the positive pressure technique
11. Resumes infusions if necessary using a new pre-prepared tubing or attaching a disinfectant cap
12. Performs hand hygiene

After the pretest, CVC management/CLABSI awareness education was conducted in the Cardiovascular Department. A posttest was given within a one to two-month post-education session. Each participant was given a pretest and a posttest with the same test number. Ensuring that each participant received the same numbered pretest and posttest unique to that person allowed the tests to be compared individually. The anonymity and privacy of all involved were prioritized throughout this project.

A pilot study was carried out to check the questionnaire for reliability. For evaluation, pilot questions were distributed to clinical instructors, nurse managers, and assistant nurse managers. All of them were familiar with the policy and procedure of central venous management and handled patients with CVC competently. Following the pilot study, some difficult questions were rephrased to provide further clarity, and some questions were discarded as they proved to be irrelevant. In this study, anonymity was ensured by not putting names on the questionnaire. Hence, the researcher would not be able to link any information to any participant.

Data collection procedure

In a staff meeting, nurse managers and cardiovascular nurses on duty were informed about the study. The participants were given 10 minutes to answer the questionnaires and return them to their clinical instructor. We ensured that an adequate number of questionnaires were handed over to the clinical instructor. The pilot study members were excluded from the actual study to avoid favoritism, as these individuals had already been exposed to the instruments used.

Data analysis

We compared the pre and posttests administered before and after the educational sessions and demonstration-based competencies. The pre and posttests contained coded demographic and content knowledge questions to maintain privacy and expedite analysis. This data was collected, documented, and analyzed. Mean scores were compared between the pre and posttests to analyze if there was an improvement. Demographic data were also coded according to the provided response. SPSS Statistics version 23 was used for data analysis to determine if learning occurred and to evaluate for percentage differences. Any missing data would be removed, and only the available data would be analyzed. The dependent variables were pretest and posttest scores related to skills and compliance. The demographic data constituted the independent variables, including age, gender, ethnicity, level of education, single or group skills, working hours, and years of experience.

## Results

We enrolled 80 participants in our study. Among them, 11.25% were under 24 years, and 3.75% were above 54 years of age, while the majority of participants (51.25%) fell under the age group of 25-34 years. Therefore, around 26.25% of nurses were 35-44 years old, and 7.50% were 45-54 years of age. There were 68 females (85%) in the cohort. The male-to-female ratio was 12:68 among the total participants. Overall, 60% (48) of all participants were Asians, 22.5% (18) were Saudi, 5% (four) were other nationalities, 11.25% (nine) were European, and one (1.25%) was American.

Most of the nurses (65%) had a bachelor's degree, while 31.25% had a master’s and 3.75% had a diploma. Among the 80 participants, 43 (53.75%) were with both pediatric and adult skills, 27.5% had adult skills, and 18.75% had pediatric skills. Most of the nurses (57.5%) worked in both day and night shifts, and only two (2.5%) nurses worked during normal office hours (0700 to 1630 hours); 26.25% were day shift nurses (0700 to 1900 hours), and 13.75% were night shift nurses (1900 to 0700 hours) (Table 5).

**Table 5:** Demographic data *Nurses who were working in different departments, either adult ward, pediatric ward, or both

Variables	Categories	N	%
Age group	19-24 years	9	11.25%
	25-34 years	41	51.25%
	35-44 years	21	26.25%
	45-54 years	6	7.50%
	Above 54 years	3	3.75%
Gender	Male	12	15%
	Female	68	85%
Ethnicity	Asian	48	60%
	European	9	11.25%
	American	1	1.25%
	Saudi	18	22.50%
	Others	4	5%
Level of education	Diploma	25	31.25%
	Bachelor’s degree	52	65%
	Master’s degree	03	53.75%
Adult or pediatric patient skills*	Only adult	22	27.50%
	Only pediatric	15	18.75%
	Both pediatric and adult	43	53.75%
Working shift	Day shift	21	26.25%
	Night shift	11	13.75%
	Both day and night shifts	46	57.50%
	Office hours	02	2.50%
Experience	Less than five years	22	27.50%
	6-10 years	30	37.50%
	11-15 years	13	16.30%
	More than 15 years	15	18.8%

Twenty-eight nurses who worked both night and day shifts belonged to the bachelor's degree group, followed by around 16 in the diploma nurses group. Nurses working the day shift mainly had bachelor's degrees (15), while six of them were diploma holders (Table 1). Of the 25 diploma holders, eight had adult skills, five had pediatric skills, and 12 had both adult and pediatric skills. Of the nurses with bachelor's degrees, 13 had adult skills, 11 had pediatric skills, and those with mixed skills were 18 in number (Table 5).

Participants with an experience of 6-10 years constituted the biggest proportion (37.5%) in the cohort in terms of work experience, and they were between 25 and 34 years of age (Table 5); 16.3% of nurses had 11-15 years of experience, 18.8% had more than 15 years of experience, and 27.5% had less than five years of experience. Most of the nurses with an experience of more than six years were Asian (Table 5). A majority of nurses with a diploma had an experience between 6-15 years and above. However, most of the nurses with a bachelor’s degree had less than five years or 5-10 years of experience (Table 6).

**Table 6:** Correlation between nurses' education and experience

		Experience			
		Less than five years	6-10 years	11-15 years	More than 15 years
Level of education	Diploma	2	8	9	6
	Bachelor’s degree	20	19	4	9
	Master’s degree	0	3	0	0

CLABSI knowledge-related pretest and posttest scores were 6.7 ±1.09 and 6.8 ±1.11, respectively. CLABSI compliance scores were 8.1 ±0.99 and 8.3 ±0.97, respectively. A slight improvement was observed in both posttests following education and training (Table 7).

**Table 7:** Assessment of CLABSI-related knowledge and compliance before and after education CLABSI: central line-associated bloodstream infection; SD: standard deviation

Tests	Mean ±SD	Median (range)
CLABSI knowledge pretest	6.7 ±1.09	7 (3-9)
CLABSI knowledge posttest	6.8 ±1.11	7 (4-10)
CLABSI compliance pretest	8.1 ±0.99	8 (5-10)
CLABSI compliance posttest	8.3 ±0.97	8 (6-10)

While we found that nurses with 6-10 years of experience achieved better scores in pre and posttest knowledge questions (p=0.01), only one nurse with more than 15 years of experience scored 10/10 in the posttest. Those nurses with an experience of fewer than five years obtained a minimum score of 3/10 on the pretest, and nurses with more than five years of experience scored 5/10 and above (Table 8).

**Table 8:** Correlation of CLABSI knowledge-related pretest and posttest scores with nurses' level of experience CLABSI: central line-associated bloodstream infection

		Pretest score							
Variable	Categories	Four	Five	Six	Seven	Eight	Nine	Ten	P-value
Experience	Less than five years	1	3	10	5	3	0	0	0.01
	6-10 years	0	2	7	13	5	3	0	
	11-15 years	0	0	5	5	2	1	0	
	More than 15 years	0	2	3	6	3	1	0	
		Posttest score							
Variable	Categories	Four	Five	Six	Seven	Eight	Nine	Ten	P-value
Experience	Less than five years	1	4	6	6	5	0	0	0.01
	6-10 years	0	2	5	13	9	1	0	
	11-15 years	0	1	4	5	3	0	0	
	More than 15 years	1	0	4	3	6	0	1	

As shown in Table 9, the 30 nurses with experience of 6-10 years and 22 nurses with experience of fewer than five years had better scores in pretest compliance tests compared to nurses with experience of more than 10 years (p=0.08).

**Table 9:** Correlation of CLABSI compliance-related pretest and posttest scores with nurses' level of experience CLABSI: central line-associated bloodstream infection

		Pretest score							
Variable	Categories	Four	Five	Six	Seven	Eight	Nine	Ten	P-value
Experience	Less than five years	0	0	1	7	7	6	1	0.08
	6-10 years	0	0	0	3	14	12	1	
	11-15 years	0	0	3	1	4	4	1	
	More than 15 years	0	0	0	1	6	6	1	
		Posttest score							
Variable	Categories	Four	Five	Six	Seven	Eight	Nine	Ten	P-value
Experience	Less than five years	0	0	3	5	7	4	3	0.03
	6-10 years	0	0	0	2	11	13	4	
	11-15 years	0	0	0	2	5	5	1	
	More than 15 years	0	0	0	2	7	5	1	

Based on the level of education, we found that nurses with a bachelor's degree scored better in pre and posttest CLABSI knowledge questions (p=0.05). For example, one nurse with a bachelor's degree scored 9/10 in the posttest, 13 nurses scored 7/10, and nine nurses scored 8/10 (Table 10).

**Table 10:** Correlation of CLABSI knowledge-related pretest and posttest scores with nurses' level of education CLABSI: central line-associated bloodstream infection

		Pretest score							
Variable	Categories	Four	Five	Six	Seven	Eight	Nine	Ten	P-value
Level of education	Diploma	1	3	10	5	3	0	0	0.08
	Bachelor’s degree	0	2	7	13	5	3	0	
	Master’s degree	0	2	8	11	5	2	0	
		Posttest score							
Variable	Categories	Four	Five	Six	Seven	Eight	Nine	Ten	P-value
Level of education	Diploma	1	4	6	6	5	0	0	0.05
	Bachelor’s degree	0	2	5	13	9	1	0	
	Master’s degree	1	1	8	8	9	0	1	

Table 11 clearly shows that nurses with a bachelor's degree and those with a diploma had better scores on the competency posttest, and five nurses with a diploma and four nurses who were degree holders scored 10/10 on the posttest (p=0.8).

**Table 11:** Correlation of CLABSI compliance pretest and posttest scores with nurses' level of education CLABSI: central line-associated bloodstream infection

		Pretest score							
Variable	Categories	Four	Five	Six	Seven	Eight	Nine	Ten	P-value
Level of education	Diploma	0	0	2	2	11	8	2	0.6
	Bachelor’s degree	0	1	2	9	18	20	2	
	Master’s degree	0	0	0	1	2	0	0	
		Posttest score							
Variable	Categories	Four	Five	Six	Seven	Eight	Nine	Ten	P-value
Level of education	Diploma	0	0	0	5	8	7	5	0.8
	Bachelor’s degree	0	0	3	6	20	19	4	
	Master’s degree	0	0	0	0	2	1	0	

Table 12 demonstrates how nurses' experience affected the ANTT standard (CVC flushing) performance. It shows that 71.6% of the participants had more than five years of experience (p=0.05), while the practical skills of nurses with a bachelor’s degree were better than those with other educational qualifications (p=0.88).

**Table 12:** Standard ANTT (CVC flushing) performance based on experience and level of education ANTT: Aseptic Non-Touch Technique; CVC: central venous catheter

Experience	ANTT standard (n=46)		P-value
	Met	Not met	
Less than five years	05 (10.8%)	5	0.05
6-10 years	13 (28.2%)	1	
11-15 years	13 (28.2%)	0	
>15 years	07 (15.2%)	2	
Education level			
Diploma	10 (21.7%)	1	0.88
Bachelor’s degree	25 (54.3%)	7	
Master’s degree	03 (6.4%)	2	

**Figure 1:**
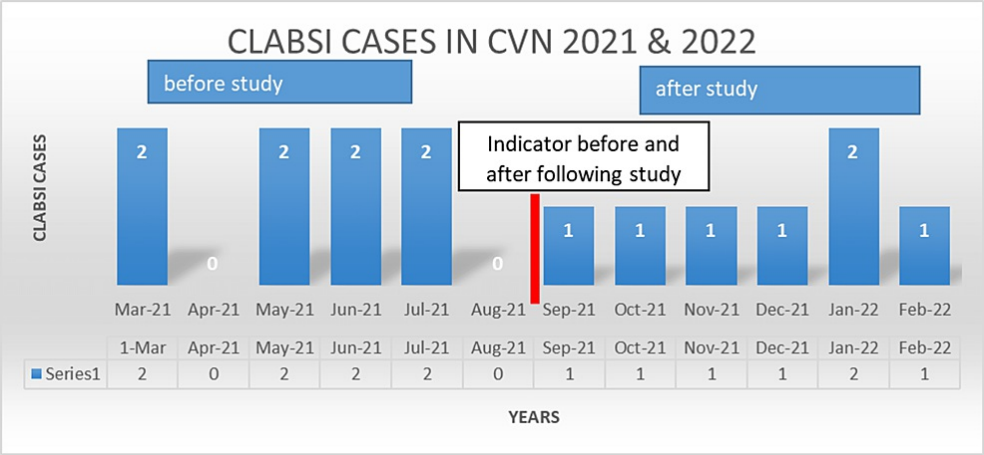
CLABSI cases in the Cardiovascular Department (Zero Harm scorecard) CLABSI: central line-associated bloodstream infection

According to the Zero Harm scorecard (2021-2022) of King Faisal Specialist Centre, Riyadh, eight cases of CLABSI were identified in the Cardiovascular Department from March 2021 to September 2021, while post-study cases were six from September 2021 to February 2022 (Figure 1).

**Figure 2:**
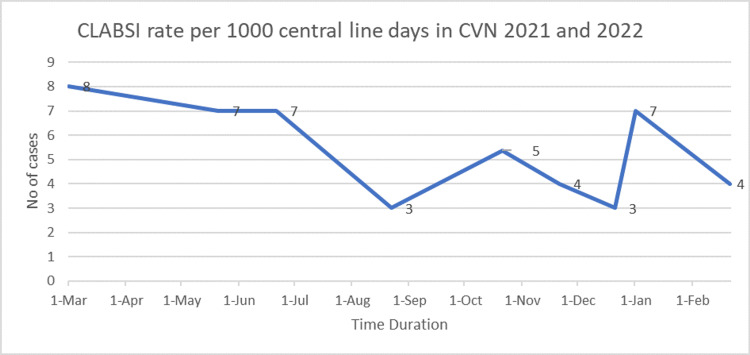
CLABSI rate per 1000 central line days in the Cardiovascular Department CLABSI: central line-associated bloodstream infection

CLABSI rate per 1000 catheter days as reported by Infection Control at King Faisal Specialist and Research Centre, Riyadh (2022) amounted to a total of 25 before and 23 after the study (Quality Department, KFSH, 2021) (Figure 2). Figure 2 demonstrates the CLABSI rates per 1000 central line days in the Cardiovascular Department from March 2021 to February 2022. The highest CLABSI rates were seen in March 2021. Post-education and training, there was a decline in the CLABSI rate in October, November, and December 2021. In January 2022, it jumped to seven but decreased to four in February 2022.

## Discussion

This study was conducted to assess nurses' knowledge and skills regarding CLABSI and methods and steps for preventing it. Based on our findings, the posttest scores for understanding and compliance improved after education sessions and training. Nurses with experience of 6-10 years had higher scores on the pretest and posttest knowledge-related questions. Nurses with less than five years of experience obtained better scores on pretest and posttest knowledge-related questions compared to nurses with experience of 11 years and above. Nurses' level of education also had a significant relationship with CLABSI pretest and posttest scores. Bachelor's degree holders obtained better scores for pre and posttest CLABSI knowledge-related questions.

However, in compliance questions, nurses with bachelor's degrees and those with diplomas obtained higher scores than those with master's degrees; therefore, the null hypothesis is accepted. Nurses with experience between 6-10 years had better competency skills in managing aseptic techniques in CVC flushing. Compliance skills were low for nurses with less than five years and more than 15 years of experience. Overall, nurses had better scores in pretest and posttest compliance questions than in pretest and posttest knowledge-related questions.

Eight nurses had a low score of 5/10 and below while showing 100% error for the second and 10th questions, which were knowledge-related questions about CVC-impregnated antibiotics for high-rate catheter-related infections and knowledge about CLABSI criteria. In addition, 87.5% gave wrong answers to questions about cleaning needleless adapters, and back and forth motion for 10 seconds. There was a 77% error in the second question for a low-scoring student, a 22% error in question seven, and an 88% error in question 10. These showed that nurses obtained knowledge post-training as the number of nurses with medium and high scores increased in terms of posttest knowledge questions. For CVC dressing change, 87.50% of nurses met the criteria, and 83.75% met the criteria for needleless adapter change, matching the compliance pretest highest scoring levels of 80%, and posttest scoring of 86%.

A similar study was done by Rasha et al. (2019) at a tertiary hospital in Saudi Arabia. Nurses with experience of 6-10 years obtained better scores in pre and posttest knowledge questions (p=0.01). Only one nurse with more than 15 years of experience scored 10/10 on the posttest (p<0.001) [5].

Moreover, the present study results are consistent with many previous studies such as those by Uba et al. (2015), Deshmukh and Shinde (2014), Pushpakala and Ravinath (2014), Bianco et al. (2013), and Meherali et al. (2010), and they strongly agree that nurse’s knowledge is influenced by professional education and training [9,12-15]. Similarly, Cooper et al. (2014) and Kim et al. (2011) have stated that educational program implementation would help reduce CLABSI and the related cost burden [16,17]. For knowledge question number 2, Chen et al.'s study (2014) supports that CVCs coated internally and externally with silver have also been shown to cause a lower incidence of catheter colonization and catheter-related bloodstream infection (CRBSI) [18].

More than half of the nurses in an area of southern Italy were female, according to a cross-sectional study. Regarding nurses' educational level, the finding corresponds with the study of El-Sol and Badawy (2017) about "a designed teaching module for preventing central line-associated bloodstream infection on ICU nurses' knowledge and practice" at Prince Mutaip-Bin Abd el Aziz hospital. They stated that nearly two-fifths of studied nurses had bachelor's degrees [19]. In an educational intervention, Acharya et al. found that educating nurses reduced CLABSI rates significantly and improved nursing knowledge scores. Education also improved compliance with hand hygiene practices by 51.75% [20]. Nurses were also more likely to follow the guidelines when there was regular ongoing training rather than a one-time-only training opportunity [21]. Furthermore, Shah et al. have explained that nurses' knowledge of care and central line maintenance improved significantly with an educational intervention. When nurses are aware of the measures necessary to reduce infections, CLABSI rates and complications are reduced. The project highlighted that the hospitals should have purposeful and deliberated CLABSI prevention efforts that go beyond the guidelines and the bundles, such as educational workshops. The benefits of these bundles are diminished if the staff is not trained to leverage the learnings. A lack of knowledge can lead to a lack of compliance with CLABSI bundles, which can reduce the quality of care the patient receives [22].

In summary, a review of current sources addressing CVC management and strategies for preventing CLABSI indicated that applying this knowledge and compliance will help minimize the incidence of CLABSI. Firstly, the benefit of this project extends to patients in non-critical care settings who undergo central line catheter insertion. Having nurses trained and equipped to provide quality care can optimize patient safety and reduce the potential for major line-related complications, including CLABSI. Secondly, the nursing staff benefitted from educational interventions, significantly improving their knowledge related to CLABSI and central line care. Evidence suggests that using multimodal approaches reduces CLABSI rates. The lack of knowledge is also a safety concern for patients, leading to an increased risk of adverse effects, rehospitalization, and potential mortality. Implementing educational interventions to reduce CLABSI can enhance patient and organizational outcomes. Finally, this project will lead to positive social change beyond patients, providers, and hospitals.

Implications

This study provides information about nurses' knowledge, skills, and compliance related to CLABSI prevention in the Cardiovascular Department. The association of nurses' experience level and education level with pre and posttest knowledge scores and work-related variables was identified. Other demographic data such as varied skill sets, working shifts, and pediatric or adult skills did not significantly correlate with knowledge, skills, compliance, and pre and posttest scores. Nurses are familiar with the central line bundle components, and their compliance scores in pre and posttests were better than those obtained for knowledge-related questions. The study by Yuling et al. (2020) suggests that care bundles can improve patients' psychological state and satisfaction related to hospitalization and decrease the length of stay; care bundles were also associated with a significant reduction in the incidence of CRBSI [23].

Limitations of the study

Although 80 nurses were recruited for the study, a larger sample size may provide better results. There was no assessment and feedback related to the long-term retention of the instructional content delivered during the training and examination. Knowledge retention after the intervention may need to be investigated over a specific period in the future. Additionally, the evaluation used only one data collection method. Assessing the impact with another measure, such as infection rates before and after the project, or participants’ behavior, such as compliance with CLABSI bundles, would have been beneficial.

Recommendations

Based on our findings in this study, we propose the following recommendations:

In order to provide staff education on central line care and the CLABSI bundle, it may be necessary to provide multiple training opportunities year-round. The relevance of this recommendation is that despite the improvement in exam scores, some nurses still did not obtain good scoring in posttest knowledge.

The educational program should be expanded to the other departments and hospital teams, such as technicians and physicians. This project focused on critical and non-critical care settings since a significant lag in knowledge had been noted. However, there is an opportunity to explore the status of knowledge and practices related to CLABSI in other units in the hospital and implement similar training opportunities for nurses in those units as well.

Sharing outcomes with the sister networks and other healthcare facilities and publishing the project outcomes can be considered as part of the effort to translate findings into practice, leading to effective measures to reduce CLABSI. Staff education is one such intervention supported by evidence to improve nurses' knowledge as well as the overall quality of care received by patients at our center and other organizations.

## Conclusions

Implementation of the educational program enhanced the nurse's knowledge, which was reflected in the improvement in the posttest scores. Staff with clinical experience of more than five years obtained higher scores in CLABSI knowledge and compliance tests. Nurses' level of education also had a significant impact on CLABSI pretest and posttest scores. In addition, the educational interventions increased the staff's knowledge of and compliance with care bundles.
